# Downregulation of CIRP Prone Cells to Oxidative Injury via Regulating Nrf2 Signaling Pathway

**DOI:** 10.1155/2022/2416787

**Published:** 2022-05-25

**Authors:** Jing Liu, Caijie Shen, Minxiao Chen, Jingjing Zhang, Meng Chen, Peng Zhong

**Affiliations:** ^1^Department of Cardiology, Ningbo First Hospital, Ningbo, Zhejiang, 315000, China; ^2^Department of Pharmacology, Renmin Hospital of Wuhan University, Wuhan 430060, China; ^3^Department of Cardiology, Renmin Hospital of Wuhan University, 430060, China; ^4^Cardiovascular Research Institute of Wuhan University, Wuhan 430060, China; ^5^Hubei Key Laboratory of Cardiology, Wuhan 430060, China; ^6^Department of Anesthesiology, Zhongnan Hospital of Wuhan University, WuhanHubei 430071, China

## Abstract

Cold-inducible RNA-binding protein (CIRP) is a cellular stress-response protein, whose expression can be induced by a variety of stress conditions. Our previous study showed that intracellular CIRP is a protective factor against cellular oxidative stress and silencing of CIRP gene prone cells to apoptosis. However, the underlying mechanism remains unknown. The present study was aimed at investigating the possible mechanisms underlying the protective role of CIRP in oxidative stress injury. Herein, we used HEK293T cells as our cell model to investigate the relation between CIRP and the possible antioxidant pathways by using the latest genetic silencing technologies. Our results showed that silencing CIRP by using SaiRNA-based genetic silencing tool leads to the downregulation of Nrf2 and Nrf2-regulated antioxidant genes in HEK293T cells. Taken together, our study identified the antioxidant Nrf2 signaling pathway as a downstream target of CIRP, and silencing CIRP may prone cells to apoptosis by downregulating the Nrf2 antioxidant pathway in response to oxidative injury.

## 1. Introduction

Cold-inducible RNA-binding protein (CIRP) was discovered 2 decades ago when studying the mechanism of cold stress adaptation in mammals [1, 2]. Since then, intensive studies suggest that CIRP is a general stress-response protein, as its expression can also be regulated by various stress conditions such as hypoxia, UV radiation, glucose deprivation, heat stress, and H_2_O_2_ [3]. In response to stress, intracellular CIRP can migrate from the nucleus to the cytoplasm and regulate mRNA stability through its binding site on the 3′-UTR of its targeted mRNAs. Through the regulation of its targets, CIRP has been implicated in multiple cellular processes such as cell proliferation, cell survival, circadian modulation, and telomere maintenance [3].

Our previous study in vitro cardiac cells showed that downregulation of CIRP could render cardiac cells prone to apoptosis in response to oxidative stress, suggesting an important role of CIRP in regulating oxidative stress injury [4]. However, the mechanism underlying this process is still unknown. Cell survival in the face of elevated oxidative stress depends on many factors, one of which is nuclear factor erythroid 2-related factor 2 (Nrf2) [5]. Nrf2 is a transcription factor that regulates the expression of many key antioxidant proteins such as heme oxygenase-1 (HO-1), NAD(P)H quinone oxidoreductase-1 (NQO-1), and other antioxidant genes. The primary role of Nrf2 signaling pathway is antioxidant stress and enhancing the protective effect of the antioxidant defense system [6]. Once the Nrf2 signaling pathway is inhibited, excessive reactive oxygen species (ROS) will be produced, which is the main factor leading to apoptosis. A very recent study suggested a possible relationship between CIRP and Nrf2 signaling pathway [7]. For example, in the neurodegenerative amyloid toxicity cell model, overexpression of CIRP was shown to prevent the elevation of ROS and increase the activities of key enzymes in antioxidant system such as superoxide dismutase (SOD), catalase (CAT), and glutathione peroxidase (GPx) [7]. Interestingly, all of these antioxidant genes are the downstream targets regulated by Nrf2 [8]. These results suggest an important role of CIRP in regulating antioxidant gene expression, and Nrf2 signaling pathway might be the possible underlying mechanism involved in the regulation of oxidative stress injury by CIRP.

In the present study, we try to investigate the possible mechanism underlying CIRP-regulated oxidative stress injury focusing on the Nrf2 antioxidant pathway in vitro HEK293T cell model. Our study identified the antioxidant Nrf2 signaling pathway as a downstream target of CIRP and possibly mediated the protective role of CIRP against oxidative stress injury.

## 2. Methods

### 2.1. Cell Culture

The HEK293T cell line was obtained from the Shanghai Institute of Biochemistry and Cell Biology (Shanghai, China). The cells were cultured in DMEM supplemented with 5% fetal bovine serum (Gibco, USA) and penicillin-streptomycin at 37°C in a humidified 5% CO_2_ atmosphere.

### 2.2. Plasmid Construction and Transfection

SaiRNA expression was initiated by the H1 promoter, with the hepatitis delta virus (HDV) ribozyme sequence at the 3′ end of the SaiRNA. BamH1/NheI sites downstream of the H1 promoter were used to insert the SaiRNA sequence. pAAV-SaiRNA vector was constructed by incorporating the SaiRNA expression elements into the pAAV-MCS backbone vector. The constructed plasmids were introduced into HEK293T cells by using polyethyleneimine (PEI) (obtained from Sigma-Aldrich).

### 2.3. RT-qPCR Analysis

RNA was isolated from the cells using TRIzol (Invitrogen Life Biotechnologies) according to the manufacturer's instructions. cDNA was synthesized using the Bio-Rad iScript Reverse Transcription Supermix (Bio-Rad). qRT-PCR analysis was performed using the Applied Biosystems VII7 (Life Technologies) for CIRP, Nrf2, HO-1, NQO-1, and *β*-actin genes using the gene-specific primers (Table 1). All primer sequences were synthesized by Qingke Biotechnology Co., Ltd. All reactions were normalized using b-actin as the endogenous control. Data were analyzed using the 2^-∆∆Ct^ method.

### 2.4. Western Blot Analysis

Total proteins were extracted from cell culture in RIPA buffer upon addition of protease inhibitors, and BCA protein assay kit (Thermo Fisher Scientific) was used to determine protein concentrations. The protein samples (40 *μ*g) were separated using the kD Mini-protean TGX precast gels (Bio-Rad) and transferred onto polyvinylidene difluoride membranes, which were then incubated with primary antibodies (Nrf2 ab137550, HO-1 (ab68477), and CIRP (ab151589), obtained from Abcam) overnight at 4°C and subsequently with secondary antibodies for 2 h at room temperature. *β*-Actin was used as an internal control to normalize gene and protein expression. *β*-Actin were obtained from Cell Signaling Technology (#4967). Chemiluminescent detection was performed using the SuperSignal West Pico Chemiluminescent Substrate (Thermo Scientific).

### 2.5. Immunofluorescence Staining

HEK293T cells plated in coverslips and grown for 24 h, followed by transfection with the target plasmid vector or blank vector, respectively, for 3 days. Then cells were treated, washed with cold PBS, fixed with PFA 4% (wt/vol) for 20 min, permeabilized with 0.1% Triton x100 in PBS, blocked with 5% milk (wt/vol), incubated with anti-Nrf2 (ab137550 from Abcam) for 1 h, washed extensively with PBS, incubated with PE-conjugated secondary antibody (#14705, from Cell Signaling Technology) for 1 h, and washed extensively. To visualize the nuclei, the cells were stained with DAPI. The fluorescence images were captured using fluorescence microscope.

### 2.6. Cell Viability Assay

Cell viability was measured with the Cell Counting Kit-8 (CCK-8) (obtained from Beyotime Biotechnology). HEK293T cells were firstly seeded in the 10 cm culture plates and then transfected with the target plasmid vector or blank vector, respectively, for 3 days. Then, these transfected HEK293T cells were distributed and seeded into a 96-well microplate and incubated for 24 h, followed by treatment with H_2_O_2_ at indicated concentrations for 24 h. Peripheral wells of microplate were filled with sterile phosphate-buffered saline (PBS). Then, 10 *μ*L of the CCK-8 solution was added to each well, and the plate was incubated for an additional 1 h. Finally, the absorbance of each well was measured by a multivolume spectrophotometer system (BioTek Instruments Inc., USA) at 450 nm.

### 2.7. Cell Apoptosis Analysis Using Flow Cytometry

HEK293T cells were transfected with the target plasmid vector or blank vector for 3 days, followed by treatment with H_2_O_2_ at 100 *μ*M for 12 h. Then Cells were stained with phycoerythrin- (PE-) conjugated Annexin V (BD Pharmingen). After washed with wash buffer, the cells were subjected to flow cytometry. The cells with both EGFP and PE positive cells were quantified to indicate the apoptotic cells.

### 2.8. Statistical Analysis

Data are represented as the mean ± SD. The Student two-tailed *t*-test was used to compare the means of two groups of samples. All statistical analysis was performed in GraphPad Pro 5.0. Statistical significance was set at *P* < 0.05.

## 3. Results

### 3.1. SaiRNA-Mediated CIRP Silencing in HEK293T Cells

**Figure 1:**
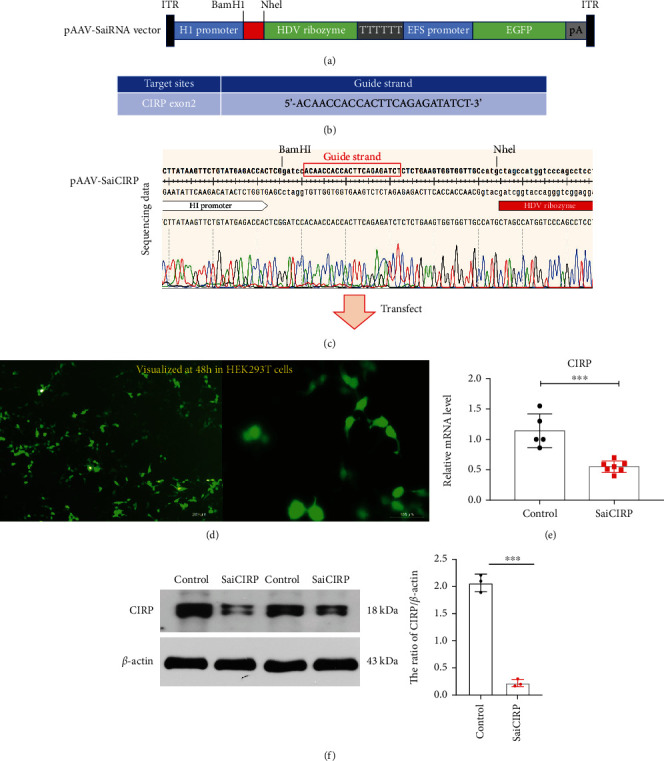
SaiRNA-mediated CIRP silencing in HEK293T cells. (a) The schematic images of the constructed pAAV-SaiRNA vector. (b) The guide sequence which targets the exon 2 of CIRP gene was used to construct pAAV-SaiCIRP vector. (c) Sanger sequencing for clone verification of the pAAV-SaiCIRP vector. (d) Represent fluorescent images of HEK293T cells after transfection of the pAAV-SaiCIRP vector at 48 hours. (e and f) The mRNA and protein expression of CIRP. HEK293T cells were transfected with pAAV-CIRP vector or pAAV-SaiRNA vector as a control for 3 days; then, these cells were subjected to RT-qPCR analysis and western blot analysis. The statistical data were presented as mean ± SD from at least three independent experiments, ^∗∗∗^*P* < 0.001.

Short-hairpin RNA (shRNA) (stem > 20 bp) has been widely used to disrupt any gene of interest; however, widespread off-target transcript silencing has been a consistent concern. Recently, an alternative shRNA design, named SaiRNA (stem < 19 bp) was shown to possess distinct processing mechanisms compared to that of shRNA and showed better on-target specificity [9]. Generally, the shRNA with stem > 20 bp will be processed into double-stranded siRNA, resulting in potential RNA interference from the guidance of either strand of the siRNA duplex. In contrast, SaiRNA with stem < 19 bp will be processed into only a single-stranded siRNA, leading to RNA interference from the guidance of the specific strand and therefore reducing the possible off-target effects of guidance by the unwanted strand of siRNA duplex [9]. Besides introducing a potent HDV ribozyme downstream of the SaiRNA, extra sequences from the 3′ end of SaiRNA can be removed and generated a short 3′ overhang, which was shown to dramatically improve SaiRNA processing and silencing activity [9]. Therefore, in the present study, we used SaiRNA design with a downstream HDV ribozyme as our gene-silencing tool. Herein, we constructed SaiRNA expression vector, called pAAV-SaiRNA vector, which contains the SaiRNA sequence cloning site and a fused HDV ribozyme downstream of the 3′ end of the SaiRNA sequence in a mammalian expression vector driven by the H1 promoter (Figure 1(a)). CIRP SaiRNA target sequence was then chosen based on a previously published shRNA sequence that can successfully silence CIRP gene expression in human cells (Figure 1(b)) [10]. Then, the target sequence was cloned into the pAAV-SaiRNA vector, and the vector incorporating our target sequence for CIRP was called pAAV-SaiCIRP. Finally, the cloned pAAV-SaiCIRP vector was sequenced at the clone sites as shown in Figure 1(c).

We then transfected HEK293T cells with pAAV-SaiCIRP. As shown in Figure 1(c), after the transfection of the cells with pAAV-SaiCIRP for 48 h in HEK293T cells, brightly green fluorescence from (EGFP) can be observed under a fluorescence microscope, suggesting the successful introduction of our target vector into cells. We then evaluate the gene silencing efficiency of the pAAV-SaiCIRP vector, and the results showed that the mRNA and protein level of CIRP can be efficiently silenced by pAAV-SaiCIRP compared to that in the control group which transfected with the blank pAAV-SaiRNA vector (Figures 1(e) and 1(f)).

### 3.2. CIRP Silencing Leads to Downregulation of the Nrf2 Signaling Pathway

The nuclear factor erythroid 2-related factor 2 (Nrf2) is a master transcriptional regulator of cellular antioxidant defense enzymes and plays an important role in regulating cellular resistance to cellular oxidative stress. As a transcriptional factor, Nrf2 can translocate into the nucleus, where it binds to the antioxidant response element (ARE) in the DNA promoter region and initiates the transcription of ARE controlled antioxidative enzymes, such as HO-1 and NQO1, which can detoxify reactive oxygen species (ROS) [11]. As our previous study showed that CIRP can regulate cell susceptibility to oxidative stress injury and downregulation of CIRP prone cell apoptosis in response to H_2_O_2_ stimulation [4], so we hypothesize that CIRP may play some role in regulating the cellular antioxidant system. Herein, we focused on our research on the relationship between CIRP and Nrf2 antioxidant pathway in HEK293T cells.

**Figure 2:**
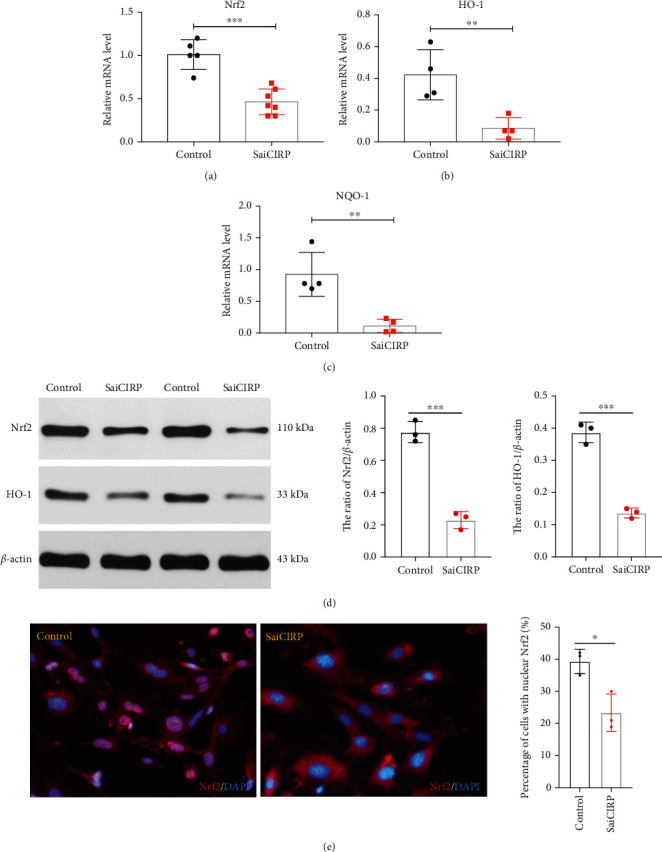
CIRP silencing leads to downregulation of the Nrf2 signaling pathway. (a–c) The mRNA of Nrf2, HO-1, and NQO-1. (d) The protein level of Nrf2 and HO-1. (e) Representative images of Nrf2 localization by using immunofluorescence staining. HEK293T cells were transfected with pAAV-CIRP vector or pAAV-SaiRNA vector as a control for 3 days; then, these cells were subjected to RT-qPCR analysis or western blot analysis or immunofluorescence staining. The statistical data were presented as mean ± SD from at least three independent experiments, *n* ≥ 3; ^∗∗^*P* < 0.01 and ^∗∗∗^*P* < 0.001.

We then detected the mRNA expression of Nrf2 and its regulated downstream antioxidant genes. Interestingly, Nrf2 and its regulated genes such as HO-1 and NQO-1 were all downregulated in CIRP-deficient cells (Figures 2(a)–2(c)). Consistently, the protein levels of Nrf2 and HO-1 were also found to be downregulated in CIRP-silenced cells (Figure 2(d)). As a transcriptional factor, Nrf2 could translocate directly to the nucleus from cytoplasm to activate gene expression. Therefore, the functional activity of Nrf2 can be evaluated by the nuclear localization of Nrf2 in the cells. Then, we further evaluated Nrf2 activity by using Nrf2 immunofluorescence staining. As shown in Figure 2(e), silencing of CIRP leads to less Nrf2 nuclear localization compared to that in the control group, suggesting that Nr2 activity was reduced in the CIRP-deficient cells. Collectively, these results suggest that Nrf2 may be a downstream target of CIRP.

### 3.3. CIRP Silencing Leads to Cells More Prone to Apoptosis in Response to Oxidative Injury

**Figure 3:**
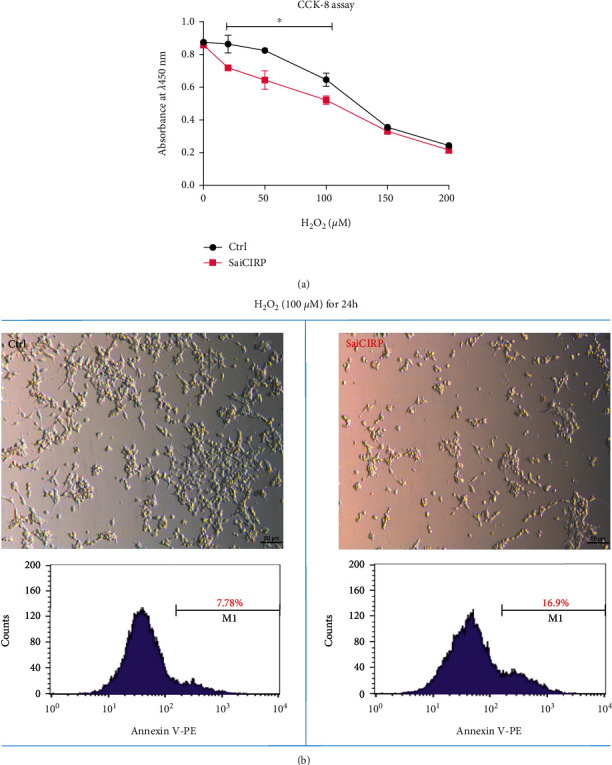
CIRP silencing leads to the cell being more prone to apoptosis in response to oxidative injury. (a) Cell survival analysis using CCK-8 assay. (b) Phase-contrast images and cells apoptosis analysis by flow cytometry with PE-conjugated-Annexin V staining. HEK293T cells were transfected with the SaiCIRP vector or control vector for 3 days, followed by the addition of H_2_O_2_ at different concentrations for another 24 hours; then, the cells were subjected to CCK8 assay or phase-contrast images analysis or stained with PE-conjugated-Annexin V and flow cytometry analysis. The statistical data were presented as mean ± SD from at least three independent experiments. *n* ≥ 3; ^∗^*P* < 0.05.

As Nrf2 is a key regulator of the antioxidant system, downregulation of the Nrf2 pathway may reduce cell antioxidant activity, thus promoting cell oxidative injury; we then tested the cell susceptibility to oxidative injury in HEK293T cells when the CIRP gene was silenced. As shown in Figure 3(a), the cells with CIRP deficiency were more prone to death in response to H_2_O_2_ treatment in HEK293T cells. Similar results were also demonstrated by phase-contrast image and cell apoptosis analysis with flow cytometry in cells stained with PE-conjugated-Annexin V (Figure 3(b)). Collectively, these results suggested that silencing of CIRP may compromise the cellular antioxidant capacity and lead the cell to be more susceptible to oxidative injury.

## 4. Discussion

CIRP is an intracellular stress-response protein that can respond to various stress conditions by changing its expression and regulating mRNA stability. Recent studies showed a protective role of CIRP in regulating cellular oxidative stress injury and cell apoptosis. In vitro experiments have shown that silencing of CIRP promotes cell apoptosis in response to H_2_O_2_ stimulation in both cardiac cells and in primary rat cortical neurons [4, 12]. In vivo study showed that overexpression of CIRP could prevent elevation of ROS induced by intracellular amyloid *β* in neural cells by decreasing the activity of oxidative biomarkers and increasing the activity of key enzymes in antioxidant systems [7]. However, the cellular mechanisms are still unclear. In the present study, we found that the Nrf2 antioxidant pathway is a downstream target of CIRP and silencing of CIRP could lead to the downregulation of Nrf2 and its regulated downstream antioxidant genes such as HO-1 and NQO-1. Therefore, downregulation of the Nrf2 antioxidant pathway may contribute to cell susceptibility to oxidative stress injury in CIRP-deficient cells.

Nrf2 is a transcription factor that coordinates the basal and stress-inducible activation of a vast array of cytoprotective genes. Nrf2 controls the expression of key components of the glutathione (GSH) and thioredoxin (TXN) antioxidant system, as well as enzymes involved in NADPH regeneration, ROS, xenobiotic detoxification, and heme metabolism, thus playing a fundamental role in maintaining the redox homeostasis of the cell [11]. Therefore, Nrf2 represents a crucial regulator of the cellular defense mechanisms against xenobiotic and oxidative stress. Previous studies have suggested a possible role of CIRP in regulating antioxidant genes. An RNA-binding protein immunoprecipitation assay in mouse testis revealed that most of the CIRP mRNA targets are associated with antioxidant activity [13]. Besides, overexpression of CIRP in neural cells was reported to upregulate key enzymes in the antioxidant system, such as superoxide dismutase (SOD), catalase (CAT), and glutathione (GPx) [7]. Interestingly, all the above antioxidant enzymes can be regulated by Nrf2 [11]. CIRP was also shown to regulate the expression of thioredoxin TXN, which is another target of Nrf2 [14]. These studies suggest a close relationship between CIRP and the antioxidant system. In the present study, by using the latest genetic technologies, we demonstrated that Nrf2 was a downstream target of CIRP, and Nrf2 may be the key connection point bridging the protective role of CIRP against oxidative injury.

Taken together, our study identified the antioxidant Nrf2 signaling pathway as a downstream target of CIRP, and silencing CIRP may prone cells to apoptosis by downregulating the Nrf2 antioxidant pathway in response to oxidative injury.

## Figures and Tables

**Table 1:** PCR primer sequence.

Gene	Species	Forward primer	Reverse primer
CIRP	Human	AGGGCTGAGTTTTGACACCAA	ACAAACCCAAATCCCCGAGAT
Nrf2	Human	CATCCAGTCAGAAACCAGTGG	GCAGTCATCAAAGTACAAAGCAT
HO-1	Human	AAGACTGCGTTCCTGCTCAAC	AAAGCCCTACAGCAACTGTCG
NQO-1	Human	GAAGAGCACTGATCGTACTGGC	GGATACTGAAAGTTCGCAGGG
*β*-Actin	Human	CTGGAACGGTGAAGGTGACA	AAGGGACTTCCTGTAACAATGCA

## Data Availability

The data used to support the findings of this study are available from the corresponding author upon request.
